# Quadriceps muscle activity during walking with a knee ankle foot orthosis is associated with improved gait ability in acute hemiplegic stroke patients with severe gait disturbance

**DOI:** 10.3389/fneur.2024.1387607

**Published:** 2024-05-07

**Authors:** Yusuke Hayashi, Kota Yamazaki, Shinya Komatsu, Naoaki Yamamoto, Shujiro Ueda, Kazunori Sato, Tomofumi Yamaguchi, Kozo Hatori, Kaoru Honaga, Tomokazu Takakura, Futoshi Wada, Akira Tanuma, Toshiyuki Fujiwara

**Affiliations:** ^1^Department of Rehabilitation Medicine, Juntendo University Graduate School of Medicine, Tokyo, Japan; ^2^Department of Rehabilitation Medicine, Juntendo University Urayasu Hospital, Chiba, Japan; ^3^Department of Physical Therapy, Faculty of Health Science, Juntendo University, Tokyo, Japan

**Keywords:** stroke, gait disorders, orthotic devices, knee ankle foot orthosis, ankle foot orthosis, gait training, electromyography

## Abstract

**Introduction:**

A knee-ankle-foot orthosis (KAFO) prevents knee buckling during walking and enables gait training for acute hemiplegic stroke patients with severe gait disturbances. Although the goal of gait training with a KAFO is to improve gait ability, that is, to acquire walking with an ankle-foot orthosis (AFO), it is not clear how gait training with a KAFO contributes to improving gait ability. Therefore, this study aimed to investigate the relationship between muscle activities during walking with a KAFO and the improvement of gait ability in hemiplegic stroke patients with severe gait disturbance.

**Methods:**

A prospective cohort study was conducted. Fifty acute hemiplegic stroke patients who could not walk with an AFO participated. Muscle activities of the paretic rectus femoris, biceps femoris, tibialis anterior, and soleus were assessed with surface electromyogram during walking with a KAFO. Electromyograms were assessed at the beginning of gait training and at the time the Ambulation Independence Measure score improved by 3 or higher, or discharge.

**Results:**

Even in patients with complete hemiplegia, paretic rectus femoris, biceps femoris, and soleus showed periodic muscle activity during walking with a KAFO. Twenty-three patients improved to an Ambulation Independence Measure score of 3 or higher and were able to walk with an AFO (good recovery group). At the beginning of gait training, paretic rectus femoris muscle activity during the first double-limb support phase was significantly higher in the good recovery group than in the poor recovery group. The rectus femoris muscle activity significantly increased from before to after acute rehabilitation, which consisted mainly of gait training with a KAFO.

**Discussion:**

For acute hemiplegic stroke patients with severe disturbance, the induction and enhancement of paretic quadriceps muscle activity during walking with a KAFO play an important role in acquiring walking with an AFO.

## 1 Introduction

Poststroke hemiparesis causes gait disturbance, which negatively affects activities of daily living (1, 2). The walking independence is one of the main goals of stroke rehabilitation. High-repetition gait training from the early stage of stroke is effective in improving gait ability in stroke patients (3–5). However, acute stroke patients with severe hemiplegia have difficulty in gait training due to severe knee buckling during the stance phase (6). A knee ankle foot orthosis (KAFO), which provides knee and ankle joint control, prevents knee buckling during the stance phase, and enables gait training for these patients (7–11). KAFOs are widely used during gait training in acute or sub-acute hemiplegic stroke patients with severe gait disturbances (7–11). However, KAFOs are difficult to use in daily life because of their large size and the difficulty of wearing them. On the other hand, ankle foot orthoses (AFOs), which provide only ankle joint control, are smaller and easier for the patient to wear. AFOs are often used by community-dwelling stroke patients with gait disturbances (12–14). Therefore, the goal of gait training with a KAFO is to enable gait training with an AFO and eventually achieve independent walking with an AFO (7–11). However, the effectiveness of gait training with a KAFO on improving gait ability has not been fully investigated (15).

In spinal cord injury patients with complete paraplegia, assisted gait training with orthoses induces paretic lower extremity muscle activity during the stance phase (16–18). In addition, repeated gait training enhances muscle activity during the stance phase and contributes to an increase in the weight-bearing capacity of the paretic lower extremity during gait training (16–18). For stroke patients with severe hemiplegia, gait training with KAFOs may also induce and then with time enhance paretic lower extremity muscle activity during gait training, and it may contribute to walking with AFOs. In fact, in subacute stroke patients with severe hemiplegia, the gluteus maximus, tensor fascia femoris, rectus femoris, semitendinosus, tibialis anterior, and medial head of gastrocnemius muscles all showed periodic muscle activity induced during stance phase (19). However, this report did not examine muscle activities separately in each gait cycle or temporal changes in muscle activity. We hypothesized that if gait training with a KAFO contributes to walking with an AFO, muscle activities involving the knee and ankle joints immobilized by the KAFO would not depend completely on the KAFO, but would be induced to resist knee buckling in the early stance phase and would be enhanced over time. To prove this hypothesis, it is necessary to examine the association between muscle activities during walking with a KAFO and the gait ability with an AFO. The Ambulation Independence Measure (AIM), a gait assessment tool we developed, is useful for assessing the gait ability with an AFO in stroke patients (11). The AIM is a measurement tool that assesses gait ability, type of orthoses, and physical assistance of stroke patients (11). A key feature of the AIM is that it limits the type of orthoses during the scoring walking test (11). Specifically, an AFO is permitted, but a KAFO or other orthoses are not allowed. An AIM score of 3 or more indicates that a KAFO is clinically unnecessary for the patient, and the patient is clinically able to convert from a KAFO to an AFO (11). In this study, this hypothesis will be examined in acute stroke patients with severe gait disturbances. These patients have severe hemiplegia and severe trunk impairment, and many of them eventually fail to achieve independent walking. Trunk function in stroke patients can be assessed with the Trunk Impairment Scale, which has proven to be highly reliable and valid (20).

Therefore, the purpose of this study was to clarify paretic lower extremity muscle activity during walking with a KAFO in hemiplegic stroke patients with severe gait disturbances and to investigate its effect on the improvement of gait ability.

## 2 Materials and methods

### 2.1 Study design

**Figure 1:**
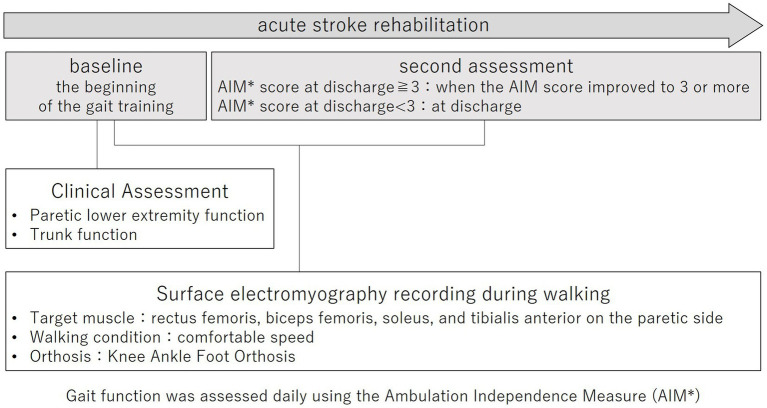
Study outline.

This prospective cohort study was conducted according to the Declaration of Helsinki and was approved by the Ethics Committee of Juntendo University Urayasu Hospital (approval number: 29-114). The purpose and procedures of this study were explained in detail, and written, informed consent was obtained from all participants. The study outline is shown in Figure 1.

### 2.2 Participants

A prospective cohort study was conducted. Participants were recruited from stroke patients within 3 days of stroke onset who were admitted to Juntendo University Urayasu Hospital in Japan from March 2018 to March 2023. The definition of acute rehabilitation initiation is often adopted within 3 days after stroke onset (21). Therefore, the participants in this study were patients who received acute rehabilitation. The diagnosis of stroke was based on each patient’s clinical history, neurological examination, and head computed tomography or magnetic resonance imaging. The inclusion criteria were as follows: (1) first unilateral hemispheric stroke; and (2) severe gait disturbance (AIM score 1 or 2) (11).

A patient with an AIM score of 1 is not able to walk with the physical assistance of one therapist using an AFO, cane, or crutch (11). A patient with an AIM score of 2 requires physical assistance to support body weight or maintain balance but shows severe knee buckling (knee flexion angle in the paretic stance phase ≥30°) during walking with an AFO, cane, or crutch (11).

The exclusion criteria were as follows: (1) inability to walk independently before onset; (2) inability to follow instructions due to various symptoms such as severe aphasia or loss of consciousness; (3) other complications or comorbidities that would alter the results of physical assessment (osteoarticular disease, recurrent stroke during hospitalization, etc.); and (4) refusal to participate in the study. A total of 50 eligible patients consented to participate in this study. The present study was conducted according to the Declaration of Helsinki and was approved by the Ethics Committee of Juntendo University Urayasu Hospital (approval number: 29-114). The purpose and procedures of this study were explained in detail, and written, informed consent was obtained from all participants.

All patients received conventional individualized inpatient rehabilitation based on the stroke rehabilitation guidelines. The physical therapy program consisted primarily of gait training with a KAFO and included range of motion training, muscle strengthening training, sitting balance training, and standing balance training as needed. The duration and frequency of physical therapy treatments ranged from 40 to 60 min per session, 5–6 times per week, depending on the patient’s physical condition.

### 2.3 Clinical assessment

Gait function was assessed daily using the AIM (score range: 1–7, best score: 7) (11). At the beginning of the gait training (baseline), paretic lower extremity motor function and trunk function were assessed. Paretic lower extremity function was assessed with the lower extremity motor scores of the Stroke Impairment Assessment Set (each score range: 0–5, best score: 5) (22). Trunk function was assessed using the Trunk Impairment Scale (score range: 0–21, best score: 21) (20).

### 2.4 Surface electromyography recording during walking

Surface electromyography (EMG) was used to evaluate paretic lower extremity muscle activity during the walking task. EMG was measured using wireless surface electromyography (Pacific Supply, Osaka, Japan). The sampling rate was 1,000 Hz. The minimum sampling rate must be at least 800 Hz (400 × 2), as specified by Nyquist’s theorem (23). Therefore, the sampling rate in this study satisfied this criterion. The target muscles were the rectus femoris, biceps femoris, soleus, and tibialis anterior on the paretic side. The target muscles were selected from the major muscles involved in the knee and ankle joints that were fixed by a KAFO. Electrode placement followed the Surface EMG for Non-Invasive Assessment of Muscles guidelines (24). Before electrode placement, the skin was rubbed with alcohol to reduce impedance.

Electromyography analysis was initially band-pass filtered at 20–250 Hz. Next, full-wave rectification was performed. The analysis section was identified based on the video synchronized with the EMG examination. The video was taken from frontal and sagittal planes. The times of initial contact and toe-off were identified from the videos using a motion analysis application (Dartfish software). If there was no clear toe-off, toe-off was defined as the time when the foot moved forward even slightly. Based on the times of initial contact and toe-off, the first double-limb support phase (1st DS), single-limb support phase (SS), second double-limb support phase (2nd DS), and swing phase (Sw) were identified (25). EMG signals were averaged for each gait phase (26). Furthermore, the same analysis was performed for the three gait cycles, and the average values for the three gait cycles were calculated. The EMG was normalized using the average value of the entire gait cycle (27).

Electromyography recording during the walking task was performed twice during hospitalization. The baseline was the beginning of gait training. The second assessment was performed when the AIM score improved to 3 or more during hospitalization. An AIM score of 3 or more indicates that a KAFO is clinically unnecessary for the patient, and the patient is clinically able to convert from a KAFO to an AFO (11). For those who did not improve to an AIM score of 3 or more during hospitalization, the second EMG measurement was performed at discharge.

### 2.5 Walking task

The walking task used for EMG recording was as follows. The walking condition was a comfortable speed. All patients used no canes or crutches. All patients used a KAFO. The knee joint control of the KAFO was fixed at 0° of knee extension using a ring-lock knee joint. The ankle joint control of the KAFO was fixed at the neutral position of dorsiflexion and plantarflexion. Assistance during walking tasks was provided by the same physical therapist familiar with gait training with a KAFO for stroke patients. The therapist assisted the patient by standing behind the patient. One of the therapist’s hands supported the patient’s anterior chest. The therapist’s other hand held the thigh cuff belt of the KAFO. Throughout the gait cycle, the therapist provided continuous balance assistance to keep the patient’s trunk in a vertical position using the hand supporting the patient’s anterior chest. During the swing phase, the physical therapist pulled up the thigh cuff belt with minimal force to assist with swinging. During the stance phase, no special assistance was provided except for continuous balance assistance throughout the entire gait cycle. For both the baseline and second assessments, the conditions during walking were the same, and the KAFO was used.

### 2.6 Statistical analysis

The relationship between the EMG during walking at baseline and improvement of gait ability was examined. An AIM score of 3 or more indicates that a KAFO is clinically unnecessary for the patient, and the patient is clinically able to convert from a KAFO to an AFO (11). Those who had an AIM score of 3 or higher in gait ability at discharge were defined as the good recovery group, whereas those who remained at an AIM score of 2 or lower in gait ability at discharge were defined as the poor recovery group. Repeated-measures two-way ANOVA (28) was performed for differences between the group (good recovery group and poor recovery group) and gait phase (1stDS, SS, 2ndDS, and Sw) in muscle activity (rectus femoris, biceps femoris, soleus, and tibialis anterior on the paretic side) during walking. If the interaction between the two factors was significant, the Bonferroni method was used to analyze significant differences between the groups in each gait phase.

The relationship between temporal EMG changes during walking and improvement of gait ability was examined. Repeated-measures two-way ANOVA (28) was performed for differences between the group (good recovery group and poor recovery group) and time (baseline and second assessments) in muscle activity during walking. The statistical analysis was performed with SPSS version 24. A *p* value <0.05 was considered significant.

## 3 Results

All 50 eligible patients could be followed up to discharge from the hospital and there were no missing values. The participants’ characteristics are shown in Table 1. The period from stroke onset to baseline was 7.3 ± 2.7 days. The length of stay was 29.6 ± 10.3 days.

**Table 1:** Patient characteristics.

Age (years)	63.1 ± 12.8
Sex (men/women)	29/21
Stroke (ischemic/hemorrhagic)	18/32
Side of lesion	19/31
Time from onset to baseline (days)	7.3 ± 2.7
Time from onset to discharge (days)	29.6 ± 10.3
Discharge destination (inpatient rehabilitation facilities/home)	50/0
SIAS-Motor hip score at baseline (score, 0–5)	1.2 ± 1.1
SIAS-Motor knee score at baseline (score, 0–5)	1.5 ± 1.4
SIAS-Motor foot score at baseline (score, 0–5)	0.7 ± 1.1
TIS at baseline (score, 0–21)	13.3 ± 4.7
AIM at baseline (1/2/3/4/5/6/7)	38/12/0/0/0/0/0
AIM at discharge (1/2/3/4/5/6/7)	16/11/12/5/5/1/0

Values are mean ± SD or number; SIAS-Motor, lower extremity motor portions of stroke impairment assessment set; TIS, Trunk impairment scale; AIM, Ambulation independence measure.

### 3.1 Relationship between muscle activity and improved gait ability

Twenty-three patients (46%) improved to an AIM score of 3 or higher and were able to walk with an AFO (good recovery group). Twenty-seven patients (54%) remained with an AIM score of 1 or 2 and were unable to walk with an AFO (poor recovery group).

**Figure 2:**
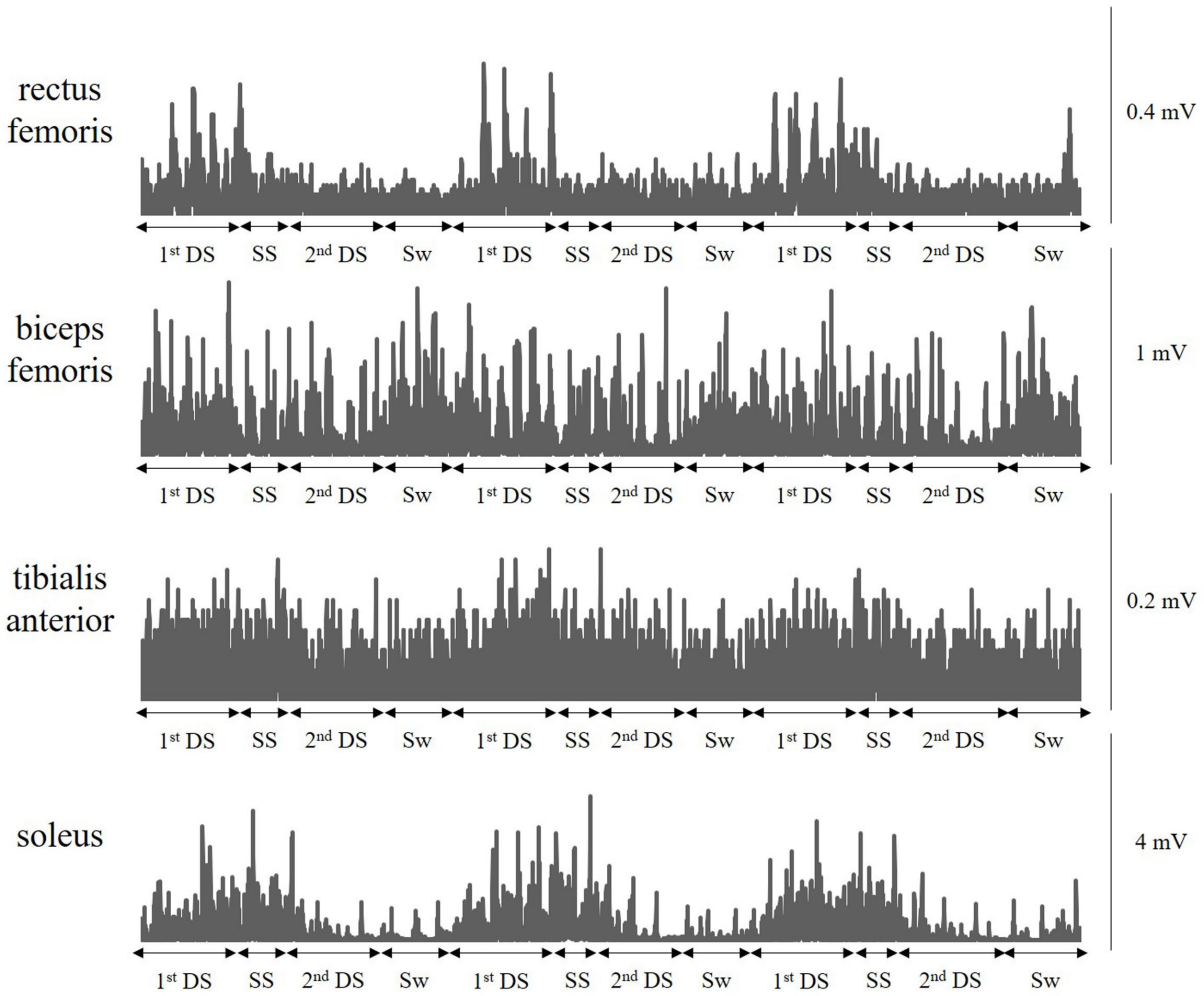
A typical muscle activity pattern during walking with a knee ankle foot orthosis. A typical muscle activity in a patient with complete hemiplegia who was unable to move his paretic lower extremity even slightly. The patient’s knee joint is fixed in 0° extension by a knee-ankle-foot orthosis, but paretic rectus femoris activity is observed in the early stance phase to resist knee buckling. This patient improved to an Ambulation Independence Measure score of 3 or more at discharge and was able to walk with an ankle-foot orthosis. 1stDS, First double-limb support phase; SS, Single-limb support phase; 2nd DS, Second double-limb support phase; and Sw, Swing phase.

Figure 2 shows typical muscle activity in a patient with complete hemiparesis (All lower extremity motor scores of the Stroke Impairment Assessment Set are 0) who was unable to move his paretic lower extremity even slightly, showing three gait cycles. Paretic rectus femoris, biceps femoris, and soleus showed periodic muscle activity. In particular, the patient’s knee joint was fixed in 0° extension by a KAFO, but paretic rectus femoris activity was observed in the early stance phase to resist knee buckling. This patient improved to an AIM score of 3 or higher at discharge (good recovery group) and was able to walk with an AFO (good recovery group).

**Figure 3:**
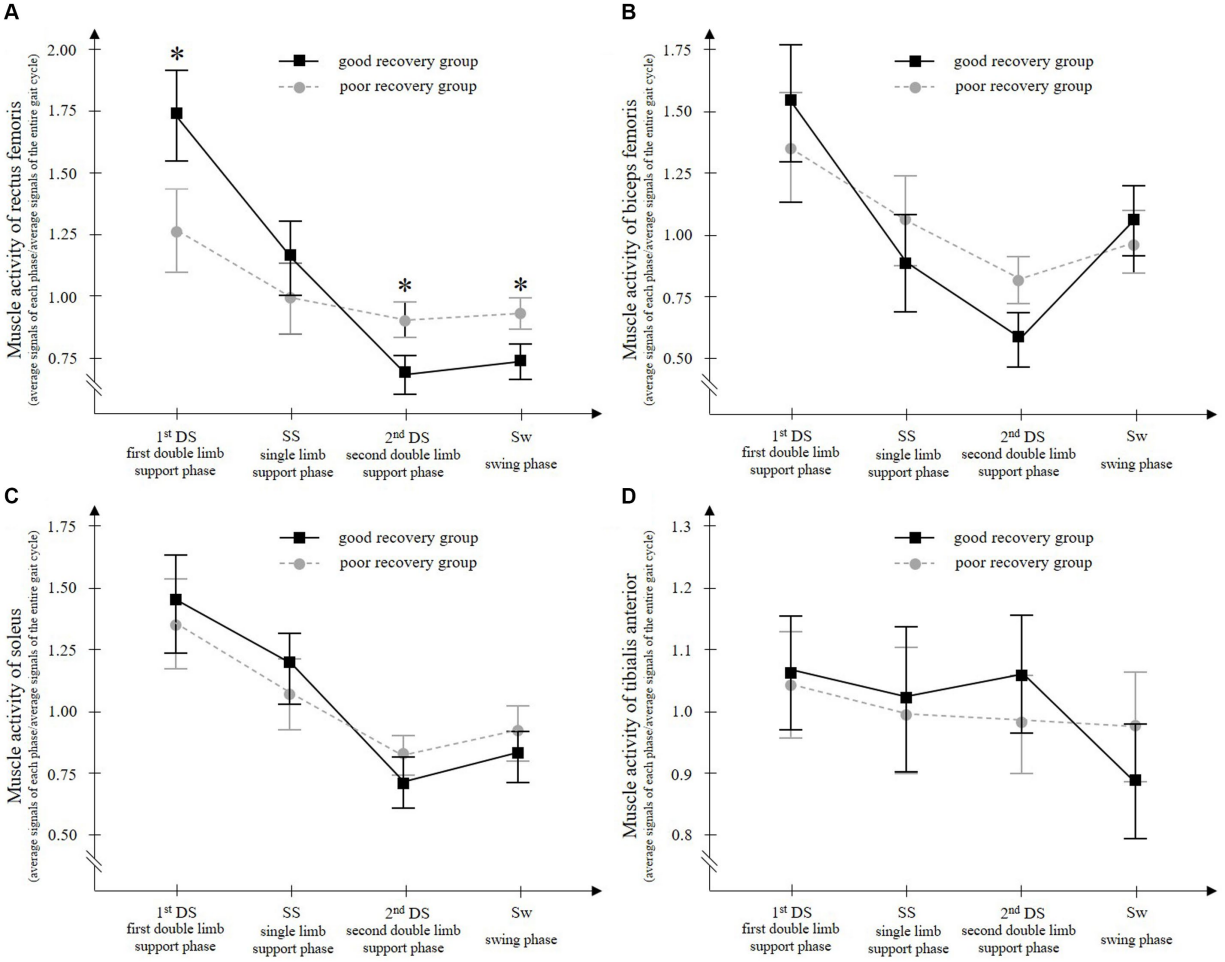
Paretic rectus femoris **(A)**, biceps femoris **(B)**, soleus **(C)**, and tibialis anterior **(D)** muscle activities during walking with a knee ankle foot orthosis. The good recovery group consisted of 23 patients who improved to an Ambulation Independence Measure score of 3 or higher at discharge and were able to walk with an ankle-foot orthosis. On the other hand, the poor recovery group consisted of 27 patients who remained with an Ambulation Independence Measure score of 2 or lower at discharge and remained unable to walk with an ankle-foot orthosis. Two-way ANOVA for the groups (good recovery group and poor recovery group) and gait cycles (the first double-limb support phase, single-limb support phase, second double-limb support phase, and swing phase) showed a significant interaction only for the paretic rectus femoris muscle. During the first double-limb support phase, the paretic rectus femoris muscle activity is significantly higher in the good recovery group than in the poor recovery group. Error bars show the 95% confidence intervals. ^*^p < 0.001 vs. poor recovery group at each gait phase.

Two-way ANOVA showed that rectus femoris muscle activity had a significant interaction between the group (good recovery group and poor recovery group) and gait phase (1stDS, SS, 2ndDS, and Sw) (*F* = 10.598, *p* < 0.001) (Figure 3). The results of the *post hoc* test using the Bonferroni method showed that the good recovery group had significantly higher paretic rectus femoris muscle activity in the 1stDS (adjusted *p* = 0.003) and significantly lower activity in the 2ndDS and Sw (both adjusted *p* < 0.001) than the poor recovery group.

Biceps femoris and soleus muscle activities had a significant main effect of the gait phase (*F* = 21.522 and 24.593, respectively, all *p* < 0.001), but no significant main effect of the group (*F* = 0.939 and 0.958, *p* = 0.338 and 0.387, respectively) and no significant interaction of the two factors (*F* = 2.262 and 0.958, *p* = 0.104 and 0.387, respectively) (Figure 3).

Tibialis anterior muscle activity showed no significant main effect of gait phase (*F* = 1.860, *p* = 0.155), no significant main effect of group (*F* = 0.320, *p* = 0.575), and no significant interaction of the two factors (*F* = 0.859, *p* = 0.464) (Figure 3).

### 3.2 Temporal changes in muscle activity

**Figure 4:**
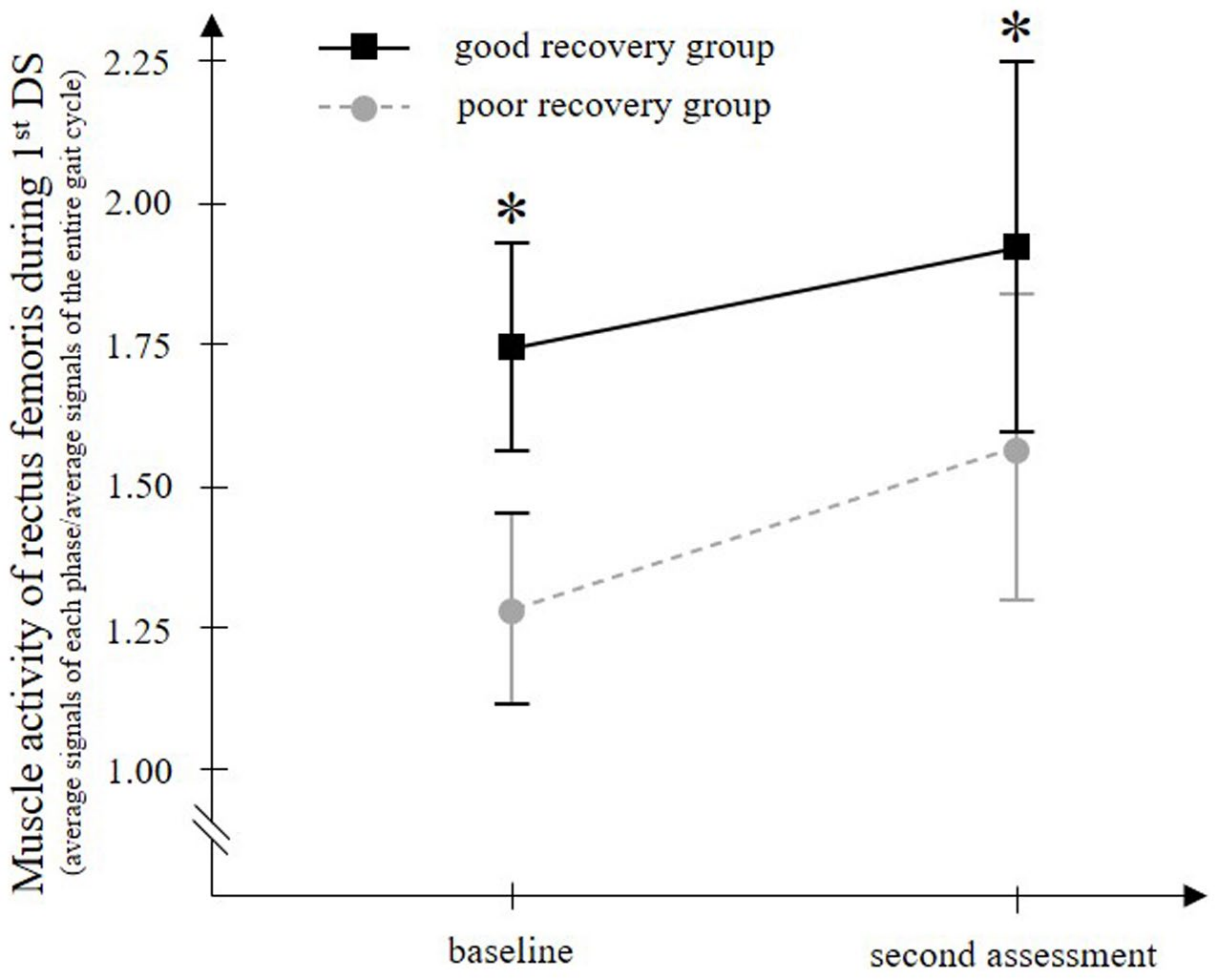
Temporal change in the paretic rectus femoris muscle activity during walking with a knee ankle foot orthosis. The good recovery group consisted of 23 patients who improved to an Ambulation Independence Measure score of 3 or higher at discharge and were able to walk with an ankle-foot orthosis. On the other hand, the poor recovery group consisted of 27 patients who remained with an Ambulation Independence Measure score of 2 or lower at discharge and remained unable to walk with an ankle-foot orthosis. In both groups, the paretic rectus femoris muscle activity in the first double-limb support phase (1st DS) is significantly increased from baseline to the second assessment. At both time points, the muscle activity is significantly higher in the good recovery group than in the poor recovery group. Error bars show the 95% confidence intervals. ^*^p < 0.001 vs. the poor recovery group at each gait phase.

For rectus femoris muscle activity in the 1stDS, which showed significant differences between groups, temporal EMG changes were compared between groups. Two-way ANOVA for paretic rectus femoris muscle activity in 1stDS showed significant main effects of time (*F* = 5.629, *p* = 0.022) and group (*F* = 802.219, *p* < 0.001), but no significant interaction of the two factors (*F* = 0.947, *p* = 0.335) (Figure 4).

## 4 Discussion

Acute hemiplegic stroke patients with severe hemiplegia have difficulty with assisted gait training with only the therapist’s assistance because of severe knee buckling during the stance phase. For these patients, KAFOs enable assisted gait training by immobilizing the knee joint in extension during the stance phase and preventing knee buckling (7, 8, 10, 19). However, the effect of gait training with a KAFO on improving gait ability has not been fully investigated (15). Therefore, in the present study, paretic lower extremity muscle activity during assisted walking with a KAFO was investigated in acute hemiplegic stroke patients with severe gait disturbances, as well as its association with the improvement of walking independence. It was found that, though the patient’s knee and ankle joints were stabilized by a KAFO, the patients were not completely dependent on the orthosis. The paretic rectus femoris, biceps femoris, and soleus muscles showed periodic muscle activity during walking. In particular, those with high rectus femoris muscle activity in the early stance phase, which is involved in knee joint control, subsequently improved their walking independence and were able to walk without a KAFO. Furthermore, the rectus femoris muscle activity in the early stance phase increased in most patients from before to after acute rehabilitation, which consisted mainly of gait training with a KAFO. These results suggest that in acute hemiplegic stroke patients with severe disturbance, the induction and enhancement of paretic quadriceps muscle activity during walking with a KAFO play an important role in acquiring walking with an AFO.

All participants in this study had an AIM score of 1 or 2 at an early stage of stroke, had severe gait disturbance, and had difficulty with assisted gait training with an AFO. All these patients underwent gait training with a KAFO. A KAFO can stabilize the patient’s knee joint in extension and enable gait training for such stroke patients (7, 8, 10, 19). The results of the present study showed that, though knee buckling was prevented by the KAFO, the patient’s resisted knee buckling in the orthosis by increasing paretic quadriceps muscle activity during the early stance phase. Furthermore, this quadriceps muscle activity was associated with improved gait ability. Within approximately 1 month of acute rehabilitation, 46% of the patients in this study improved their AIM score to 3 or higher and were able to walk with an AFO. On the other hand, 54% remained at an AIM score of 1 or 2 and were unable to walk with an AFO. One of the differences related to walking recovery is the paretic rectus femoris muscle activity pattern during walking with a KAFO. Patients with improved gait ability (AIM score 3 or more) showed higher paretic rectus femoris muscle activity during the early stance phase at early stroke onset, compared to patients with unchanged gait ability (AIM score 1 or 2). An AFO does not have a mechanism to control the knee joint. To achieve a high AIM score, particularly three or more, patients must control their knee joints during walking. The difference between an AIM score of 2 and 3, which was used for a KAFO to an AFO conversion in the present study, was whether the peak knee joint flexion angle was greater than or less than 30 degrees during the stance phase of walking with an AFO (11). In normal subjects, rapid flexion of the knee joint is observed during the early stance phase, because the ground reaction force passes behind the knee joint (29). This rapid knee flexion is controlled by the eccentric contraction of the quadriceps muscle, resulting in a maximum knee flexion angle of 20° or less (29). We believe that quadriceps muscle activity during the early stance phase plays an important role in hemiplegic stroke patients with severe disturbances, as in healthy subjects. At the beginning of gait training, all patients had difficulty controlling their knee joints, and this may have been caused by insufficient quadriceps muscle activities during the early stance phase of walking due to severe hemiplegia. Gait training with a KAFO induces paretic quadriceps muscle activity in the early stance phase and enhances it through acute rehabilitation. Those with relatively high paretic quadriceps muscle activities during walking from early onset would have reached sufficient muscle activity to prevent knee buckling within the acute rehabilitation period, allowing them to control the knee joint and walk with an AFO. On the other hand, those with relatively low paretic quadriceps muscle activities during walking in early onset would have had difficulty reaching sufficient muscle activity to prevent knee buckling during the acute rehabilitation period, and would have remained unable to walk with an AFO. For these patients, it may be necessary to perform gait training with a longer-term perspective or to add more specialized training to increase paretic quadriceps muscle activity during the early stance phase of walking. Specifically, body weight support treadmill gait training with electrical stimulation and robotic devices (30) would be expected to lead to improvement. Pedaling exercises (31, 32) that can be easily applied to non-ambulatory patients may also be useful.

### 4.1 Limitation

A limitation of this study is that no control group was used and the direct effect of gait training using a KAFO on the improvement of muscle activity and gait function could not be examined. Another limitation is that muscle activity was assessed during assisted walking, and muscle activity was influenced by the method of assistance and gait speed, the training time and frequency were not identical, the amount of gait training could not be quantitatively assessed, the observation period was short, and the final gait ability could not be confirmed. In this study, during the stance phase, no special assistance was provided, except for continuous balance assistance throughout the entire gait cycle, but in body weight support treadmill gait training using a robotic device, quantitative evaluation was possible by adjusting levels of bodyweight support and guidance force. However, the number of facilities that can provide gait training using special equipment is limited. Therefore, we believe that this study is highly valuable because it demonstrates the effect of gait training with a KAFO, which can be easily performed in clinical settings. In the future, a randomized controlled trial should be conducted to clarify whether gait training with a KAFO contributes to the enhancement of quadriceps muscle activity during the early stance phase and to the improvement of gait ability.

### 4.2 Conclusion

Among hemiplegic stroke patients with severe gait disturbances who had difficulty walking with an AFO, 46% were able to acquire walking with an AFO and 54% were unable to acquire walking with an AFO throughout the acute rehabilitation phase. One of the differences between the two groups was the paretic quadriceps muscle activity during walking with a KAFO. The patients who were able to acquire walking with an AFO had significantly higher quadriceps muscle activity during the early stance phase than those who did not. In addition, both groups showed a significant increase in this quadriceps muscle activity throughout the acute rehabilitation phase. Thus, the induction and enhancement of paretic quadriceps muscle activity during walking with a KAFO may play an important role in the acquisition of walking with an AFO.

## Data availability statement

The raw data supporting the conclusions of this article will be made available by the authors, without undue reservation.

## Ethics statement

The studies involving humans were approved by the Ethics Committee of Juntendo University Urayasu Hospital. The studies were conducted in accordance with the local legislation and institutional requirements. The participants provided their written informed consent to participate in this study.

## Author contributions

YH: Conceptualization, Data curation, Formal Analysis, Investigation, Methodology, Project administration, Resources, Software, Supervision, Validation, Visualization, Writing – original draft, Writing – review & editing. KY: Conceptualization, Formal Analysis, Investigation, Methodology, Project administration, Software, Validation, Visualization, Writing – review & editing. SK: Formal Analysis, Investigation, Methodology, Software, Validation, Visualization, Writing – review & editing. NY: Formal Analysis, Investigation, Methodology, Software, Validation, Visualization, Writing – review & editing. SU: Formal Analysis, Investigation, Methodology, Software, Validation, Visualization, Writing – review & editing. KS: Formal Analysis, Investigation, Methodology, Software, Validation, Visualization, Writing – review & editing. TY: Data curation, Formal Analysis, Methodology, Visualization, Writing – review & editing. KHa: Data curation, Formal Analysis, Methodology, Visualization, Writing – review & editing. KHo: Data curation, Formal Analysis, Methodology, Visualization, Writing – review & editing. TT: Data curation, Formal Analysis, Methodology, Visualization, Writing – review & editing. FW: Data curation, Formal Analysis, Methodology, Visualization, Writing – review & editing. AT: Data curation, Formal Analysis, Methodology, Visualization, Writing – review & editing. TF: Conceptualization, Data curation, Formal Analysis, Funding acquisition, Methodology, Project administration, Resources, Software, Supervision, Validation, Visualization, Writing – review & editing.

## References

[1] Patel AT Duncan PW Lai SM Studenski S. The relation between impairments and functional outcomes Poststroke. Arch Phys Med Rehabil. (2000) 81:1357–63. doi: 10.1053/apmr.2000.9397 11030501

[2] Reding MJ Potes E. Rehabilitation outcome following initial unilateral hemispheric stroke. Life Table Analys Approach Stroke. (1988) 19:1354–8. doi: 10.1161/01.str.19.11.1354, PMID: 3188120

[3] Hornby TG Holleran CL Hennessy PW Leddy AL Connolly M Camardo J. Variable intensive early walking Poststroke (views): a randomized controlled trial. Neurorehabil Neural Repair. (2016) 30:440–50. doi: 10.1177/1545968315604396, PMID: 26338433

[4] Klassen TD Dukelow SP Bayley MT Benavente O Hill MD Krassioukov A. Higher doses improve walking recovery during stroke inpatient rehabilitation. Stroke. (2020) 51:2639–48. doi: 10.1161/strokeaha.120.029245, PMID: 32811378

[5] Moore JL Nordvik JE Erichsen A Rosseland I Bø E Hornby TG. Implementation of high-intensity stepping training during inpatient stroke rehabilitation improves functional outcomes. Stroke. (2020) 51:563–70. doi: 10.1161/strokeaha.119.027450 31884902 PMC7034641

[6] Miyamoto S Ogasawara K Kuroda S Itabashi R Toyoda K Itoh Y. Japan stroke society guideline 2021 for the treatment of stroke. Int J Stroke. (2022) 17:1039–49. doi: 10.1177/17474930221090347, PMID: 35443847 PMC9615334

[7] Abe H Nishiyama K Yamamoto Y Okanuka T Yonezawa Y Matsumoto K. Impact of alternate gait training using knee-ankle-foot orthoses with oil damper ankle hinge in patients with subacute severe hemiplegia. Brain Sci. (2021) 11:1430. doi: 10.3390/brainsci11111430, PMID: 34827429 PMC8615545

[8] Fujii R Sugawara H Ishikawa M Fujiwara T. Effects of different orthoses used for gait training on gait function among patients with subacute stroke. Prog Rehabil Med. (2020) 5:20200023. doi: 10.2490/prm.20200023, PMID: 33029567 PMC7533285

[9] Kakurai S Akai M. Clinical experiences with a convertible thermoplastic knee-ankle-foot orthosis for post-stroke hemiplegic patients. Prosthetics Orthot Int. (1996) 20:191–4. doi: 10.3109/03093649609164442, PMID: 8985999

[10] Yamanaka T Akashi K Ishii M. Stroke rehabilitation and long leg brace. Top Stroke Rehabil. (2004) 11:6–8. doi: 10.1310/g8rf-312l-g6fw-a8jw, PMID: 15480948

[11] Hayashi Y Yamazaki K Takeda K Ueda S Mikawa S Hatori K. The development of ambulation Independence measure: a new measurement tool to assess gait ability in acute stroke patients. Neurorehabilitation. (2022) 50:409–16. doi: 10.3233/nre-210289, PMID: 35068419 PMC9277666

[12] Dogan A Mengulluoglu M Ozgirgin N. Evaluation of the effect of ankle-foot orthosis use on balance and mobility in hemiparetic stroke patients. Disabil Rehabil. (2011) 33:1433–9. doi: 10.3109/09638288.2010.533243, PMID: 21091133

[13] Erel S Uygur F Engin Simsek I Yakut Y. The effects of dynamic ankle-foot orthoses in chronic stroke patients at three-month follow-up: a randomized controlled trial. Clin Rehabil. (2011) 25:515–23. doi: 10.1177/0269215510390719, PMID: 21285288

[14] Daryabor A Arazpour M Aminian G. Effect of different designs of ankle-foot orthoses on gait in patients with stroke: a systematic review. Gait Posture. (2018) 62:268–79. doi: 10.1016/j.gaitpost.2018.03.026, PMID: 29587246

[15] Kobayashi E Hiratsuka K Haruna H Kojima N Himuro N. Efficacy of knee-ankle-foot orthosis on functional mobility and activities of daily living in patients with stroke: a systematic review of case reports. J Rehabil Med. (2022) 54:jrm00290. doi: 10.2340/jrm.v54.87, PMID: 35582910 PMC9285852

[16] Dietz V. Evidence for a load receptor contribution to the control of posture and locomotion. Neurosci Biobehav Rev. (1998) 22:495–9. doi: 10.1016/s0149-7634(97)00035-3, PMID: 9595560

[17] Dietz V Müller R Colombo G. Locomotor activity in spinal man: significance of afferent input from joint and load receptors. Brain. (2002) 125:2626–34. doi: 10.1093/brain/awf273, PMID: 12429590

[18] Dietz V Colombo G Jensen L Baumgartner L. Locomotor capacity of spinal cord in paraplegic patients. Ann Neurol. (1995) 37:574–82. doi: 10.1002/ana.410370506, PMID: 7755351

[19] Abe H Kadowaki K Tsujimoto N Okanuka T. A narrative review of alternate gait training using knee-ankle-foot orthosis in stroke patients with severe hemiparesis. Phys Ther Res. (2021) 24:195–203. doi: 10.1298/ptr.R0015, PMID: 35036252 PMC8752868

[20] Fujiwara T Liu M Tsuji T Sonoda S Mizuno K Akaboshi K. Development of a new measure to assess trunk impairment after stroke (trunk impairment scale): its psychometric properties. Am J Phys Med Rehabil. (2004) 83:681–8. doi: 10.1097/01.phm.0000137308.10562.20, PMID: 15314532

[21] de Aquino M Miranda J Mendes Borges V Bazan R José Luvizutto G Shinosaki SM. Early mobilization in acute stroke phase: a systematic review. Top Stroke Rehabil. (2023) 30:157–68. doi: 10.1080/10749357.2021.2008595, PMID: 34927568

[22] Chino N Sonoda S Domen K Saitoh E Kimura A. Stroke impairment assessment set (Sias). Jpn J Rehabil Med. (1994) 31:119–25. doi: 10.2490/jjrm1963.31.119

[23] Merletti R. Standards for reporting EMG data. J Electromyogr Kinesiol. (1999) 9:III-IV.

[24] Hermens HJ Freriks B Disselhorst-Klug C Rau G. Development of recommendations for Semg sensors and sensor placement procedures. J Electromyogr Kinesiol. (2000) 10:361–74. doi: 10.1016/s1050-6411(00)00027-4, PMID: 11018445

[25] Akl AR Gonçalves P Fonseca P Hassan A Vilas-Boas JP Conceição F. Muscle co-activation around the knee during different walking speeds in healthy females. Sensors. (2021) 21:1430. doi: 10.3390/s21030677, PMID: 33498231 PMC7863926

[26] Modica JR Kram R. Metabolic energy and muscular activity required for leg swing in running. J Appl Physiol. (2005) 98:2126–31. doi: 10.1152/japplphysiol.00511.2004, PMID: 15894536

[27] Yang JF Winter DA. Electromyographic amplitude normalization methods: improving their sensitivity as diagnostic tools in gait analysis. Arch Phys Med Rehabil. (1984) 65:517–21. PMID: 6477083

[28] Armitage P Berry G Matthews JNS. Statistical Methods in Medical Research. 4th ed. Oxford, UK: Blackwell Science (2002).

[29] Perry J. Gait Analysis: Normal and Pathological Function. 2nd ed. Thorofare, NJ: Slack Inc. (2010).

[30] Lin J Hu G Ran J Chen L Zhang X Zhang Y. Effects of bodyweight support and guidance force on muscle activation during Locomat walking in people with stroke: a cross-sectional study. J Neuroeng Rehabil. (2020) 17:5. doi: 10.1186/s12984-020-0641-6, PMID: 31931825 PMC6958616

[31] Tanuma A Fujiwara T Yamaguchi T Ro T Arano H Uehara S. After-effects of pedaling exercise on spinal excitability and spinal reciprocal inhibition in patients with chronic stroke. Int J Neurosci. (2017) 127:73–9. doi: 10.3109/00207454.2016.1144055, PMID: 26785780

[32] Lee K. Emg-triggered pedaling training on muscle activation, gait, and motor function for stroke patients. Brain Sci. (2022) 12:76. doi: 10.3390/brainsci12010076, PMID: 35053819 PMC8773827

